# Longitudinal change in mismatch negativity (MMN) but not in gamma-band auditory steady-state response (ASSR) is associated with psychological difficulties in adolescence

**DOI:** 10.1093/cercor/bhad346

**Published:** 2023-10-10

**Authors:** Kaori Usui, Kenji Kirihara, Tsuyoshi Araki, Mariko Tada, Daisuke Koshiyama, Mao Fujioka, Ryoichi Nishimura, Shuntaro Ando, Shinsuke Koike, Hiroshi Sugiyama, Toru Shirakawa, Rie Toriyama, Mio Masaoka, Shinya Fujikawa, Kaori Endo, Syudo Yamasaki, Atsushi Nishida, Kiyoto Kasai

**Affiliations:** Department of Neuropsychiatry, Graduate School of Medicine, The University of Tokyo, Tokyo, 113-8655, Japan; Department of Community Mental Health & Law, National Institute of Mental Health, National Center of Neurology and Psychiatry, Tokyo, 187-8551, Japan; Department of Neuropsychiatry, Graduate School of Medicine, The University of Tokyo, Tokyo, 113-8655, Japan; Disability Services Office, The University of Tokyo, Tokyo, 113-8655, Japan; Department of Neuropsychiatry, Graduate School of Medicine, The University of Tokyo, Tokyo, 113-8655, Japan; Department of Neuropsychiatry, Teikyo University Hospital, Mizonokuchi, Tokyo, 213-8507, Japan; Department of Neuropsychiatry, Graduate School of Medicine, The University of Tokyo, Tokyo, 113-8655, Japan; Office for Mental Health Support, Center for Research on Counseling and Support Services, The University of Tokyo, Tokyo, 113-8655, Japan; The International Research Center for Neurointelligence (WPI-IRCN), University of Tokyo Institutes for Advanced Study (UTIAS), The University of Tokyo, Tokyo, 113-0033, Japan; Department of Neuropsychiatry, Graduate School of Medicine, The University of Tokyo, Tokyo, 113-8655, Japan; Department of Neuropsychiatry, Graduate School of Medicine, The University of Tokyo, Tokyo, 113-8655, Japan; Department of Neuropsychiatry, Graduate School of Medicine, The University of Tokyo, Tokyo, 113-8655, Japan; Department of Neuropsychiatry, Graduate School of Medicine, The University of Tokyo, Tokyo, 113-8655, Japan; Department of Psychiatry and Behavioral Sciences, Tokyo Metropolitan Institute of Medical Science, Tokyo, Japan; Department of Neuropsychiatry, Graduate School of Medicine, The University of Tokyo, Tokyo, 113-8655, Japan; The International Research Center for Neurointelligence (WPI-IRCN), University of Tokyo Institutes for Advanced Study (UTIAS), The University of Tokyo, Tokyo, 113-0033, Japan; University of Tokyo Institute for Diversity & Adaptation of Human Mind (UTIDAHM), Tokyo, 113-8655, Japan; Center for Evolutionary Cognitive Sciences, Graduate School of Art and Sciences, The University of Tokyo, Tokyo, 153-8902, Japan; Department of Neuropsychiatry, Graduate School of Medicine, The University of Tokyo, Tokyo, 113-8655, Japan; Department of Neuropsychiatry, Graduate School of Medicine, The University of Tokyo, Tokyo, 113-8655, Japan; Department of Electrical Engineering and Computer Science, Faculty of Systems Design, Tokyo Metropolitan University, Tokyo, 192-0397 Japan; Department of Neuropsychiatry, Graduate School of Medicine, The University of Tokyo, Tokyo, 113-8655, Japan; Department of Neuropsychiatry, Graduate School of Medicine, The University of Tokyo, Tokyo, 113-8655, Japan; Department of Neuropsychiatry, Graduate School of Medicine, The University of Tokyo, Tokyo, 113-8655, Japan; Department of Psychiatry and Behavioral Sciences, Tokyo Metropolitan Institute of Medical Science, Tokyo, Japan; Department of Psychiatry and Behavioral Sciences, Tokyo Metropolitan Institute of Medical Science, Tokyo, Japan; Department of Psychiatry and Behavioral Sciences, Tokyo Metropolitan Institute of Medical Science, Tokyo, Japan; Department of Neuropsychiatry, Graduate School of Medicine, The University of Tokyo, Tokyo, 113-8655, Japan; The International Research Center for Neurointelligence (WPI-IRCN), University of Tokyo Institutes for Advanced Study (UTIAS), The University of Tokyo, Tokyo, 113-0033, Japan; University of Tokyo Institute for Diversity & Adaptation of Human Mind (UTIDAHM), Tokyo, 113-8655, Japan

**Keywords:** adolescence, mismatch negativity (MMN), gamma-band auditory steady-state response (ASSR), psychological difficulties

## Abstract

Adolescence is a critical period for psychological difficulties. Auditory mismatch negativity (MMN) and gamma-band auditory steady-state response (ASSR) are representative electrophysiological indices that mature during adolescence. However, the longitudinal association between MMN/ASSR and psychological difficulties among adolescents remains unclear. We measured MMN amplitude for duration and frequency changes and ASSR twice in a subsample (*n* = 67, mean age 13.4 and 16.1 years, respectively) from a large-scale population-based cohort. No significant longitudinal changes were observed in any of the electroencephalography indices. Changes in SDQ-TD were significantly associated with changes in duration MMN, but not frequency MMN and ASSR. Furthermore, the subgroup with higher SDQ-TD at follow-up showed a significant duration MMN decrease over time, whereas the subgroup with lower SDQ-TD did not. The results of our population neuroscience study suggest that insufficient changes in electroencephalography indices may have been because of the short follow-up period or non-monotonic change during adolescence, and indicated that the longitudinal association with psychological difficulties was specific to the duration MMN. These findings provide new insights that electrophysiological change may underlie the development of psychosocial difficulties emerging in adolescence.

## Introduction

Adolescence, the period between childhood and adulthood, is a crucial stage for the emergence of psychological difficulties. In this period, adolescents tend to have heightened emotional reactivity, impulsive behavior, sensitivity to peer influence, and exploratory activation because they are limited in their ability to engage in self-regulation to override these emotions and actions (Spear 2013). This is also the peak period for the onset of most mental disorders (Kessler et al. 2005). The vulnerability to mental disorders is paralleled by neurobiological changes (Casey et al. 2010; Lee et al. 2014). Therefore, understanding the neurodevelopmental trajectory associated with psychological difficulties is a priority for biological psychiatry.

Human neuroimaging studies have shown the dynamic development of adolescent brains, such as cortical thinning (Giedd et al. 1999; Tamnes et al. 2017), white matter volume increase (Paus 2010), and changes in functional connectivity (Fair et al. 2009). Brain developmental changes are likely to be attributed to putative changes in neurotransmitter levels, such as the major excitatory and inhibitory neurotransmitters—glutamate and gamma-aminobutyric acid (GABA, Ghisleni et al. 2015). Studies on adolescent rodents and primates have shown functional expression of the subunit in glutamatergic N-methyl-D-aspartate (NMDA) receptor (Flores-Barrera et al. 2014), increased parvalbumin expression on GABAergic neurons (Caballero et al. 2014), and structural changes in GABA receptor subunits (Hashimoto et al. 2008) in the frontal lobe. Maturation of these neurotransmitter systems is thought to promote the excitatory/inhibitory (Excitatory/Inhibitory, E/I) balance on neurons and within networks, contributing to functional maturation and synaptic pruning (Selemon 2013; Caballero et al. 2016), which is crucial for brain maturation. Human studies using magnetic resonance spectroscopy to investigate regional levels of glutamate and GABA in healthy adolescents have reported a decreasing tendency of glutamate and an increase of GABA with age in adolescents (Silveri et al. 2013; Ghisleni et al. 2015). However, few studies have addressed this issue using electrophysiological measurements such as electroencephalography (EEG), which directly reflects neuronal activity.

Auditory mismatch negativity (MMN) and auditory steady-state response (ASSR), which are EEG measures, are considered markers of glutamatergic and GABAergic neurotransmission function (Tada et al. 2019, 2020). MMN is an event-related potential (ERP), usually measured in a passive oddball task. Several types of MMN depending on the paradigm of the oddball task are present, and the typical types include duration MMN (dMMN) and frequency MMN (fMMN), which use stimuli that deviate in sound duration and frequency. Because the antagonist of the NMDA receptor attenuates the MMN amplitude (Javitt et al. 1996; Umbricht et al. 2000; Rosburg and Kreitschmann-Andermahr 2016), MMN is thought to be a marker of glutamatergic neurotransmission function. ASSR is an electrophysiological response entrained to the frequency and phase of auditory stimuli. ASSR is maximal in the gamma-band frequency range in humans (Galambos et al. 1981), and ASSR around 40 Hz (40 Hz-ASSR) is a highly reproducible index in clinical studies (Tada et al. 2020). Because synaptic interactions between parvalbumin-positive GABAergic interneurons and pyramidal neurons generate gamma oscillations (Cardin et al. 2009; Sohal et al. 2009), ASSR is thought to be an index of GABAergic neurotransmission function. Therefore, MMN and 40 Hz-ASSR are expected to be biomarkers that reflect important neurotransmitter functions related to adolescent neurodevelopment.

Several previous studies have reported that MMN and ASSR dynamically change during adolescence. Cross-sectional studies have reported that the MMN amplitude increases with age (7–12 vs. 13–16 vs. 35–56 years, Bishop et al. 2011), whereas another study reported no significant changes (Mahajan and McArthur 2015). For gamma-band ASSR, age-related increases have been shown in cross-sectional studies in age groups of 5–52 years (Rojas et al. 2006) and 19–45 years (Poulsen et al. 2007) and in a longitudinal study from 10 to 11.5 years of age (Poulsen et al. 2009). Another adolescent cross-sectional study observed an inverted U-shaped development trajectory (Cho et al. 2015). However, longitudinal studies have been scarce, and no study has examined changes in MMN and ASSR simultaneously in a longitudinal cohort study of adolescent population-based samples.

The current study aimed to investigate the longitudinal associations of MMN and ASSR with psychological difficulties in healthy mid-adolescents recruited from the Tokyo TEEN Cohort (TTC, Ando et al. 2019), a large longitudinal general population-based birth cohort (*n* = 3171) in the Tokyo metropolitan area. We hypothesized that developmental changes in MMN and ASSR are associated with changes in psychological difficulties during adolescence.

## Materials and methods

### Experimental design

This was a longitudinal study examining the association between MMN, ASSR, and psychological difficulties. Study participants were those who took part in the population-neuroscience in Tokyo Teen Cohort (pn-TTC, Okada et al. 2019) study described below during the entry period of 2016–2018. We obtained measures at 2 time points—baseline (time 1) and at ~2–3 years of follow-up (time 2)—and examined the association between changes in EEG measures and change in psychological difficulties from times 1 to 2.

### Participants

The current study was performed as part of the pn-TTC (*n* = 301), a longitudinal study exploring the neurobiological substrates of development during adolescence (Okada et al. 2019). Participants in the pn-TTC were subsampled from a larger cohort study entitled TTC (Ando et al. 2019). As part of the first wave of the pn-TTC, we obtained subsamples of 10-year-old adolescents and their primary parents ~1 year after their participation in the TTC and conducted longitudinal biennial follow-up surveys. EEG measurements were started from the second wave of the pn-TTC. The Strengths and Difficulties Questionnaire (SDQ) was rated close to EEG measurements. We also collected a history of mental disorders for the parents in the TTC study and the 10-year-old adolescents who participated, as well as present illnesses for adolescents by checking all present illnesses on the day close to the EEG measurements. In the current study, we defined the second wave of pn-TTC as the baseline (time 1) completed in 2018 and the third wave of pn-TTC as the follow-up (time 2) completed in 2020. Written informed assent was obtained from each participant, and written informed consent was obtained from their primary parent before participation. All protocols were approved by the research ethics committees of the Faculty of Medicine at the University of Tokyo (approval nos. 629, 10057, and 10069), the Tokyo Metropolitan Institute of Medical Science (approval nos. 12–35), and the Graduate University for Advanced Studies (SOKENDAI; approval no. 2012002).

The pn-TTC participants were demographically and socioeconomically representative of the TTC study population (Okada et al. 2019). Although the inclusion criteria of the TTC study were not explicitly stated, subjects born between September 2002 and August 2004 along with their parents living in the 3 Tokyo municipalities were randomly selected and those who agreed to participate in our study were enrolled. The exclusion criteria of the pn-TTC study (i–v) and for measuring EEG (vi) were as follows: (i) current problems in mental health, interpersonal relationships, or behaviors, (ii) visual or hearing disabilities, (iii) past history of head injury accompanied by more than 5 min of loss of consciousness, (iv) current chronic endocrine disease or metabolic disease, (v) current use of medications that affect the central nervous system, and (vi) auditory impairment revealed by audiometer testing in both ears at a 30-dB sound pressure level and a tone frequency of 1,000 Hz and 40 dB at 4,000 Hz. The detailed methods for participant recruitment and the rationales have been described in our previous studies (Ando et al. 2019; Okada et al. 2019).

### Evaluation of psychological status

We used the Japanese version of the SDQ (Goodman 1997; Moriwaki and Kamio 2014) to assess the psychological status, which was rated by the primary parent. The SDQ includes 5 subscales. The total difficulties (TD) score was calculated as the sum of 4 subscales (emotional symptoms, conduct problems, hyperactivity/inattention, and peer problems; higher scores indicate higher difficulties). For the prosocial behavior subscale, a higher score indicated higher strength. We used the TD score as a measure of psychological difficulty in the subsequent analyses.

### M‌MN

We used 2 standard auditory oddball paradigms with duration- and frequency-deviant stimuli. For dMMN, standard tones (1,000 Hz, 50 ms) were 1,800 (90%) of the stimuli, and deviant tones (1,000 Hz, 100 ms) were 200 (10%) of the stimuli. For fMMN, standard tones (1,000 Hz, 50 ms) were 1800 (90%) of the stimuli, and deviant tones (1200 Hz, 50 ms) were 200 (10%) of the stimuli. The stimuli settings were the same as those in our previous studies (Koshiyama et al. 2018a; Fujioka et al. 2020). The order of the 2 paradigms was counterbalanced across participants. All stimuli were presented binaurally through earphones, whereas the participants watched a silent movie. The auditory parameters were delivered at an 80 dB sound pressure level, 1 ms rise/fall time, and 500 ms stimulus-onset asynchrony.

### Gamma-band ASSR

Auditory steady-state stimuli were click sounds (1 ms) presented in 500-ms trains at 20 and 40 Hz. Click sound trains were presented at each frequency in a single block of 200 trains. The stimuli settings were the same as those used in our previous studies (Tada et al. 2016; Koshiyama et al. 2018b).

### E‌EG recording and analyses

EEG data were recorded using an EEG acquisition system with active electrodes (Polymate II, AP2516; Miyuki Giken, Tokyo, Japan), which is compact with a maximum of 16 channels. EEG data were acquired at Fz and Cz and referenced to the left mastoid. The ground electrode was located on the right mastoid. Vertical electrooculograms were recorded from the electrodes above and below the right eye. The sampling rate was set at 1,000 Hz, with the analog filter bandpass set at 0.05–333 Hz. To confirm the arousal level during the task, we measured the subjective sleepiness between each task using the Japanese version of the Stanford Sleepiness Scale (Hoddes et al. 1972, 1973). Supplementary Table 1 shows the sleepiness index immediately after each EEG task.

The data were analyzed using Vision Analyzer (version 2.1, Brain Products, Munich, Germany). To analyze the MMN response, the signals were digitally filtered at 0.1–30 Hz. Epochs were extracted from −100 to 500 ms for MMN analysis and from −250 to 750 ms for ASSR analysis. The mean of the pre-stimulus baseline was subtracted from the baseline correction for the MMN analysis. Eyeblink artifacts were corrected using the Gratton & Coles method (Gratton et al. 1983). We excluded epochs exceeding ±50 μV for the MMN and ± 75 μV for the ASSR. After artifact rejection, we excluded data that included less than half of the epochs in the standard and deviant condition of the MMN and ASSR tasks. The amplitudes at Fz were used for analysis in accordance with previous studies because the MMN and ASSR responses were robust around the Fz (Corcoran et al. 2018; Koshiyama et al. 2021).

To analyze dMMN and fMMN, we obtained ERP waveforms for both standard and deviant stimuli through across-trial averaging. The MMN waveform was obtained as the average waveform by subtracting the responses to standard stimuli from the responses to deviant stimuli. The MMN latency in adolescents is less known than that in adults, and it is not consistent across studies (Cheour et al. 2001; Bishop et al. 2011; Murphy et al. 2013). Therefore, we decided to use the data-driven mean amplitude around the grand average waveform as the MMN value. Following inspection of the grand average waveforms of MMN, peak detection was used to identify MMN latency from 100 to 250 ms at the Fz channel, where the MMN amplitude was maximal. The peak latency of the grand average MMN waveform was calculated for each MMN type and period (time 1 or 2). Furthermore, the amplitude was defined as the mean amplitude between 25 ms before and after the peak in the grand average.

To analyze ASSR, we performed time-frequency analyses with a short-term Fourier transformation and then calculated event-related spectral perturbation (ERSP) and intertrial phase coherence (ITC) as indices of ASSR using EEGLAB (version 2.1, Swartz Center for Computational Neuroscience, California, USA; Delorme and Makeig 2004). ERSP indicates event-related changes in power relative to the pre-stimulus baseline. ITC indicates phase consistency across trials and ranges between 0 (random phase across trials) and 1 (identical phase across trials). Decreases in ERSP and ITC reflect reduced neural response to auditory steady-state stimulation. We calculated the mean ERSP and ITC by averaging the data over the stimulation time (0–500 ms) and frequency (36–45 Hz). We used the ERSP and ITC data of 40-Hz ASSR for subsequent analyses.

### Procedure

Of the 301 participants in the pn-TTC study, 131 underwent EEG measurement at time 1. Of these, 12 were excluded because of low measurement quality and equipment problems. A total of 119 participants remained at time 1. Of these, 37 did not participate in the follow-up measurement (time 2), 8 were excluded because of low measurement quality and equipment problems, and the remaining 11 were excluded because of missing SDQ scores. Finally, 67 participants (33 girls) were included in the analysis.

### Statistical analysis

All analyses were performed using SPSS version 27 (IBM Corp., NY, USA). For the comparison of EEG indices and SDQ-TD scores between times 1 and 2, we performed paired *t*-tests. Bonferroni corrections for multiple comparisons were applied (statistically significant level was set at *P* < 0.01). Although SDQ-TD was the main outcome measure of interest in the current study, we compared scores on the SDQ subscales at times 1 and 2 using paired *t*-tests as an exploratory analysis. In addition, to test whether there was any selection bias for follow-up data, we compared the demographics, EEG indices, and SDQ-TD between participants with and without follow-up using independent *t*-tests. Independent *t*-tests were also performed to examine sex differences in EEG indices (values of times 1 and 2, and changes) as part of the exploratory analysis.

A multiple regression analysis using a stepwise method was performed to examine the relationships between changes in EEG indices and SDQ-TD scores. Each value of change was calculated by subtracting the value at time 1 from the value at time 2. Changes in the SDQ-TD score were used as the dependent variable, and changes in dMMN amplitude, fMMN amplitude, ASSR ERSP, ASSR ITC, age in months at SDQ assessment, follow-up period at SDQ assessment (months), and sex (as a dummy variable; girls, 0; boys, 1) were used as independent variables. The significance threshold for analysis was set at *P* < 0.05. Although SDQ-TD was the main outcome measure of interest in the current study, multiple regression analyses with a stepwise method were conducted for the association between the change in dMMN amplitude, sex, and follow-up period and change in SDQ subscales.

### Post hoc analysis

As the main results indicated that changes in dMMN amplitudes over time were associated with changes in SDQ-TD scores, we conducted a post hoc analysis to confirm and further investigate this association. We divided the participants into high and low SDQ-TD groups based on the median SDQ-TD scores at time 2. The 2-way analysis of variance (ANOVA) was then conducted with group (high and low SDQ-TD at time 2) as the between-subject factor and time (times 1 and 2) as the within-subject factor. When a group-by-time interaction was significant, the subsequent within-group tests at the specified timepoints (times 1 and 2) were performed using paired *t*-tests, and between-group tests at these timepoints were performed using independent *t*-tests. The significance threshold for ANOVA was set at *P* < 0.05, and Bonferroni corrections for multiple comparisons were applied for subsequent tests (statistically significant level was set at *P* < 0.0125).

## Results

**Fig. 1:**
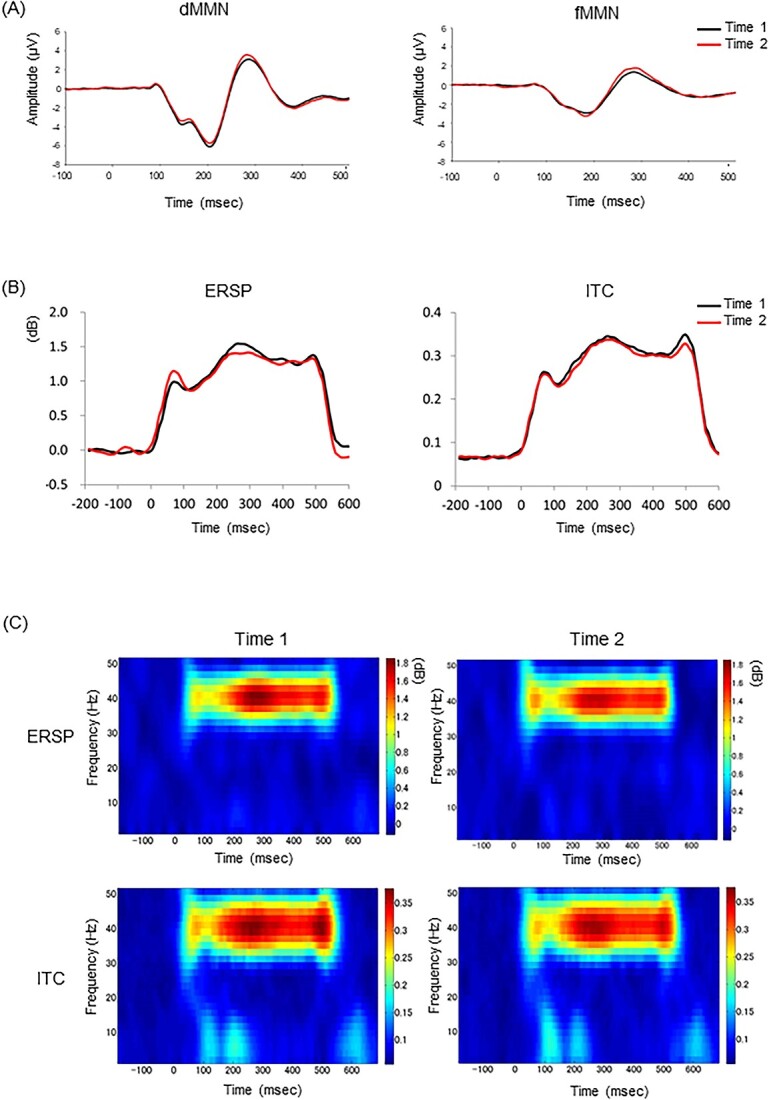
Average waveforms of MMN, time-course of ASSR, and time-frequency maps of ASSR at times 1 and 2. A) The average waveforms (*n* = 67) for amplitude (μV) of dMMN (left) and fMMN (right) at Fz at time 1 (colored in black) and time 2 (red). B) The time-course of the ERSP (left) and the ITC (right) and indices of the ASSR at Fz at time 1 (black) and time 2 (red). C) The grand average time-frequency maps for ERSP and ITC at the Fz are described in times 1 and 2. The color bar indicates the ERSP (dB)/ITC at each time-frequency point.

The demographics, EEG indices, and SDQ-TD scores at times 1 and 2 are presented in Table 1. Regarding the longitudinal EEG data, the grand average waveforms of the dMMN and fMMN and the time-course and grand average time-frequency maps for the ITC/ERSP of ASSR are shown in Fig. 1, and detailed EEG measurement data are shown in Supplementary Table 1. In addition, the grand average waveforms, including MMN, as well as standard and deviant conditions, are shown in Supplementary Fig. 1. As part of an exploratory analysis, we compared the demographics, EEG indices, and SDQ-TD between participants with and without follow-up to confirm whether there was any selection bias for follow-up data (Table 2), and no significant differences were found. We also compared sex differences of EEG indices at times 1 and 2, and the changes between these time points, but no significant differences were observed between sexes (Supplementary Table 2).

**Table 1:** Alterations in MMN, ASSR, and SDQ-TD scores between times 1 and 2.

	Time 1 Mean ± SD	Time 2 Mean ± SD	Statistics (Paired *t*-tests)
*N* (sex ratio boys/girls)	67 (34/33)		N.A.
Age (years) at EEG measurement	13.4 ± 0.5 (range: 12.3–14.3)	16.1 ± 0.8 (range: 14.4–18.2)	—^a^
Age (years) at SDQ assessment	13.3 ± 0.8 (range: 12.3–14.8)	15.5 ± 0.7 (range: 14.2–17.4)	—^a^
*N* (%) of subjects with mental disorders	0 (0%)^b^	1 (1.5%)	N.A.
*N* (%) of parents with mental disorders	1 (1.5%)		N.A.
dMMN amplitude (μV)	−5.05 ± 2.67	−4.81 ± 2.09	*t*_66_ = −0.94, *P* = 0.35^c^
fMMN amplitude (μV)	−2.68 ± 1.87	−2.96 ± 1.90	*t*_66_ = 1.06, *P* = 0.29^c^
ASSR ERSP (dB)	1.15 ± 1.22	1.14 ± 1.36	*t*_66_ = 0.03, *P* = 0.98^c^
ASSR ITC	0.29 ± 0.12	0.28 ± 0.13	*t*_66_ = 0.37, *P* = 0.71^c^
SDQ-TD	6.2 ± 4.1	6.6 ± 4.8	*t*_66_ = −0.85, *P* = 0.40 ^c^

Abbreviations: N.A., not applicable; SDQ, Strengths and Difficulties Questionnaire; dMMN, duration mismatch negativity; fMMN, frequency mismatch negativity; ASSR, auditory steady-state response; ERSP, event-related spectral perturbation; ITC, intertrial phase coherence; SDQ-TD, SDQ-total difficulties score.

^a^Paired *t*-tests were not performed because the age difference between the 2 times in the same participant was self-evident.

^b^Total *n* = 62.

^c^The statistical significance level was set at *P* < 0.01 (Bonferroni correction).

**Table 2:** Comparison of demographic data between participants who were followed up and those without follow-up.

	Participants followed up Mean ± SD (range)	Participants without follow-up Mean ± SD (range)	Statistics
*N* (sex ratio M/F)^a^	67 (34/33)	52 (30/22)	*χ^2^* = 0.57, df = 1, *P* = 0.45
Age (years) at EEG assessment^b^	13.4 ± 0.5 (12.3–14.3)	13.6 ± 0.5 (12.6–14.6)	*t*_117_ = 1.95, *P* = 0.054
Age (years) at SDQ assessment^b^	13.3 ± 0.8 (12.3–14.8)	13.4 ± 0.5 (12.5–14.6)	*t*_117_ = 1.67, *P* = 0.098
*N* (%) of subjects with mental disorders	0 (0%)^c^	1 (2.2%)^d^	N.A.
*N* (%) of parents with mental disorders	1 (1.5%)	1 (1.9%)	N.A.
dMMN amplitude (μV)^b^	−5.05 ± 2.67	−5.7 ± 2.34	*t*_117_ = −1.40, *P* = 0.16
fMMN amplitude (μV)^b^	−2.68 ± 1.87	−2.9 ± 2.04	*t*_117_ = −0.61, *P* = 0.54
ASSR ERSP (dB)^e^	1.15 ± 1.22	1.81 ± 1.94	*t*_81.16_ = 2.13, *P* = 0.04^f^
ASSR ITC^e^	0.29 ± 0.12	0.33 ± 0.15	*t*_93.27_ = 1.54, *P* = 0.13
SDQ-TD^b^	6.2 ± 4.1	6.3 ± 3.8	*t*_117_ = 0.18, *P* = 0.86

Abbreviations: EEG, electroencephalography; SDQ, Strengths and Difficulties Questionnaire; dMMN, duration mismatch negativity; fMMN, frequency mismatch negativity; ASSR, auditory steady-state response; ERSP, event-related spectral perturbation; ITC, intertrial phase coherence; TD, total difficulties; SD, standard deviation.

^a^A chi-square test was performed.

^b^Independent *t*-tests were performed.

^c^Total *n* = 62.

^d^Total *n* = 46.

^e^Welch’s test with adjusted degrees of freedom was performed because equal variance was not assumed. Bonferroni corrections were not applied because of the exploratory nature of the analysis.

^f^ *P* < 0.05.

### Longitudinal change of MMN, ASSR, and SDQ-TD between times 1 and 2

A comparison of the EEG indices and the SDQ-TD between times 1 and 2 is presented in Table 1. There were no significant differences in MMN amplitudes, ASSR ERSP/ITC, and SDQ-TD (*P* > 0.01). For supplemental information, the results of the comparisons between the SDQ subscales at times 1 and 2 are presented in Supplementary Table 3.

### Association between longitudinal changes in EEG indices and psychological difficulties

**Fig. 2:**
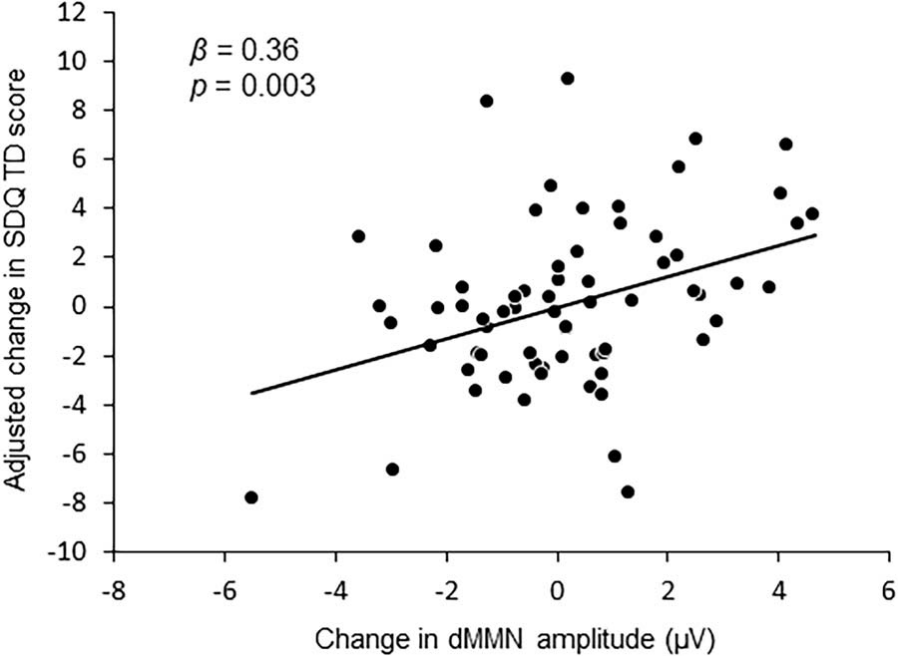
Correlation between change in dMMN amplitude and adjusted change in SDQ-TD. Scatter plots and regression line. The horizontal axis represents the change in amplitude (μV) of dMMN, and the vertical axis represents the adjusted change in the total difficulties score of the strengths and difficulties questionnaire (SDQ-TD). The adjusted changes in SDQ-TD were calculated by subtracting Beta_(sex)_^*^sex, Beta_(follow-up period)_^*^follow-up period, and Beta_(intercept)_ from the change in the SDQ-TD based on the results of multiple regression analysis.

We performed multiple regression analyses to investigate the association between longitudinal changes in EEG indices and the SDQ-TD (Table 3). Changes in dMMN amplitude (*β* = 0.36, *P* = 0.003), sex (*β* = −0.29, *P* = 0.011; the TD score was worse for girls than for boys), and follow-up period (*β* = 0.24, *P* = 0.04) were significant regressors of change in the SDQ-TD score (adjusted *R*^2^ = 0.17, *P* = 0.002). Figure 2 shows the correlation between changes in dMMN amplitude and the adjusted changes in SDQ-TD. The adjusted changes in SDQ-TD were calculated by subtracting Beta_(sex)_^*^sex, Beta_(follow-up period)_^*^follow-up period, and Beta_(intercept)_ from the change in the SDQ-TD score. For supplemental information, the results of the association between the change in dMMN amplitude, sex, and follow-up period and change in SDQ subscales, using multiple regression analyses with a stepwise method, are presented in Supplementary Table 4.

**Table 3:** Association between change in SDQ-TD and change in dMMN amplitude.

Step	*β*	SE	*F*	Adjusted *R*^2^	*P*
*Change in SDQ-TD*			5.61	0.17	0.002^b^
Change in dMMN amplitude	0.36	0.20			0.003^b^
Sex (Girls, 0; Boys, 1)	−0.29	0.80			0.011^a^
Follow-up period	0.24	0.06			0.04^a^

^a^ *P* < 0.05.

^b^ *P* < 0.01.

Abbreviations: SDQ-TD, Strengths and Difficulties Questionnaire-total difficulties score; dMMN, duration mismatch negativity.

### Post hoc analysis of the comparison of dMMN amplitude between the high and low SDQ-TD groups

**Fig. 3:**
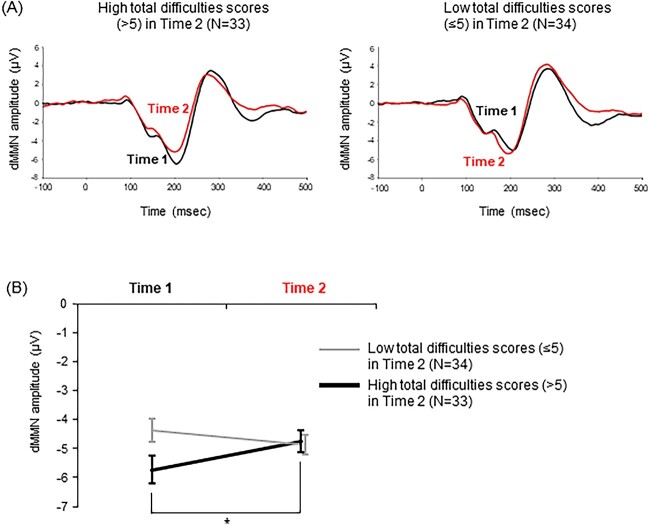
Change of dMMN between times 1 and 2 in the groups with high and low TD scores at time 2. A) The average waveforms for dMMN in the group with high TD scores (>5) at time 2 (*n* = 33; left) and in the group with low TD scores (≤5) at time 2 (*n* = 34; right) at the Fz in time 1 (colored in black) and time 2 (red). B) The results of subsequent tests in dMMN amplitude, showing the interaction of group (high or low TD group) and time (times 1 and 2). The *y*-axis indicates dMMN amplitude (μV). The black or gray bar indicates data of the group with high TD scores or the group with low TD scores, respectively. Error bars represent standard errors. ^^*^^Paired *t*-tests between groups at time 1: *t*_32_ = −2.77, *P* = 0.009.

Since the median SDQ-TD score at time 2 (*n* = 67) was 5, we divided the participants into 2 groups: a high TD group (SDQ-TD > 5, *n* = 33) and a low TD group (SDQ-TD ≤ 5, *n* = 34). The ANOVA showed a significant group-by-time interaction (*F* (1, 65) = 10.07, *P* = 0.002). There was no main effect of group (*F* (1, 65) = 1.40, *P* = 0.24) or time (*F* (1, 65) = 1.11, *P* = 0.30). The subsequent paired *t*-tests indicated that the group with a high SDQ-TD at time 2 showed a significant decline in the dMMN amplitude at time 2 relative to time 1 (*t*_32_ = −2.77, *P* = 0.009), whereas the group with a low SDQ-TD at time 2 did not (*t*_33_ = 1.63, *P* = 0.11; Fig. 3). Additionally, the subsequent independent *t*-tests indicated that the dMMN amplitude was not significantly different between the high and low TD group at time 1 (*t*_65_ = −2.14, *P* = 0.04; the threshold for statistical significance was set at *P* < 0.0125) or time 2 (*t*_65_ = 0.21, *P* = 0.84).

## Discussion

This is the first study to investigate the longitudinal relationship between MMN plus 40 Hz-ASSR and psychological difficulties during adolescence in a population-based cohort. No significant longitudinal changes in dMMN and fMMN amplitudes, ASSR indices (ERSP/ITC), or SDQ-TD scores were observed. Individuals with decreased dMMN amplitude over time, female sex, and longer follow-up duration showed increased SDQ-TD scores in the linear regression model.

In the entire group, MMN amplitudes and ASSR values did not show significant developmental changes over 2–3 years during mid-adolescence. Several studies reported that MMN amplitude increased with age during adolescence (Oades et al. 1997; Bishop et al. 2011). These insufficient changes in MMN in our study may have been because of the short follow-up period. In addition, previous studies reported that the ASSR trajectory was an inverted-U shape, with increases from childhood to adolescence and subsequent decreases toward adulthood (Cho et al. 2015). The lack of significant changes in the ASSR in our study may be because of non-monotonic increases or decreases during adolescence.

We found a significant association between the decrease in dMMN amplitude over time and an increase in the SDQ-TD score. Furthermore, we found that longitudinal dMMN changes in the group with higher psychological difficulties differed significantly from those in the lower group. The dMMN changes were spread over a wide range, about ±6 μV. However, the variance of these changes did not deviate from previous findings of variances in dMMN amplitudes observed in multiple measurements within 2–3 weeks for the research of test–retest reliability (Wang et al. 2021). The MMN reduction over time in the high SDQ-TD group is compatible with the findings of the study by Laurens et al. (2020), where they observed the developmental trajectory of MMN during adolescence in a group with antecedents of schizophrenia. Compared with the increased MMN amplitude trajectory in typically developing children aged 9–16 years, in the group with antecedents of schizophrenia, an initial small increase became a pronounced reduction later during adolescence. Our study showed individual differences in the developmental change of MMN, even in the general population of adolescents without psychopathology, and that the change differences were associated with general psychological difficulties. Since the onset of mental disorders such as schizophrenia typically occurs during adolescence and young adulthood (Paus et al. 2008), further decreased MMN during adolescence may be associated with the onset of mental disorders later. It is necessary to clarify the association between the MMN development trajectory and the onset of psychiatric disorders over a longer term in order to test these hypotheses.

Furthermore, changes in MMN associated with psychological difficulties may reflect adolescent brain development. During adolescence, increased connectivity in the frontal and temporal areas occurs in association with changes in MMN (Cooray et al. 2016). In addition, at the level of neurotransmission, MMN is decreased with the glutamatergic NMDA receptor antagonists, suggesting that MMN may reflect the impaired function of glutamatergic neurotransmission (Javitt et al. 1996; Tada et al. 2019). Therefore, the atypical development of mainly frontal and temporal circuits or glutamatergic neurotransmission function may be involved in the background of increased psychological difficulties. It is expected that basic nonhuman studies will clarify the neural basis involved in MMN development during adolescence.

Of the EEG indices, only changes in dMMN amplitudes were significantly associated with changes in the SDQ-TD scores. Previous studies have suggested that dMMN and fMMN amplitudes are different electrophysiological indices in terms of sensitivity or function. Some studies reported a higher sensitivity of dMMN than fMMN during a comparison between healthy individuals and those with an ultra-high risk state for psychosis (UHR, Fujioka et al. 2020) or future conversion of UHR (Bodatsch et al. 2011). Furthermore, while fMMN amplitude was related to neurocognitive function, dMMN amplitude was related to the impairment of global or social function in youth with UHR (Kim et al. 2014; Carrion et al. 2015; Koshiyama et al. 2018a). Since the SDQ-TD score is a global scale for social adaptation and mental health in adolescence (Goodman 1997), the scores might be correlated with the dMMN amplitude in our study. In addition, the lack of a significant relationship between ASSR and psychological difficulties may have been influenced by the nonlinear developmental change in ASSR (Cho et al. 2015).

Our study has certain strength and limitations. Our “population-neuroscience” (Okada et al. 2019; Kasai et al. 2022) study design that measured neuroscientific indices in individuals recruited from a large-scale population-based cohort may have an advantage in reducing the effect of selection bias. The limitation is that changes in EEG indices may be influenced by other factors besides adolescent developmental changes. Furthermore, our measurement period was restricted to 2 sessions during middle adolescence. As we could not determine the trajectory of the EEG indices during the entire period of adolescence, further longitudinal follow-up of the sample is required, which is currently ongoing.

In conclusion, the results of our population neuroscience study found that atypical developmental changes in dMMN are associated with psychological difficulties emerging in adolescence. It may help to clarify the biological basis behind the psychological difficulties that occur in the general adolescent population.

## Supplementary Material

supplementary_information_TTCeeg_CerebCortex_usui_230904_bhad346

## Data Availability

Due to the ethical restriction, the data cannot be made open access to the public. However, the data may be available from the corresponding author upon reasonable request and through the ethical committee's approval.
