# Supplementary material for: Longitudinal change in mismatch negativity (MMN) but not in gamma-band auditory steady-state response (ASSR) is associated with psychological difficulties in adolescence
Source: Cereb Cortex. 2023 Oct 10;33(22):11070–9. doi: 10.1093/cercor/bhad346 (PMC10631957; doi:10.1093/cercor/bhad346)
Supplement: supplementary_information_TTCeeg_CerebCortex_usui_230904_bhad346 [file supplementary_information_ttceeg_cerebcortex_usui_230904_bhad346.docx]

**Supplementary Material**

**Supplementary Figure** Average waveforms of standard, deviant, and MMN condition in dMMN and fMMN at Time 1 and Time 2

**
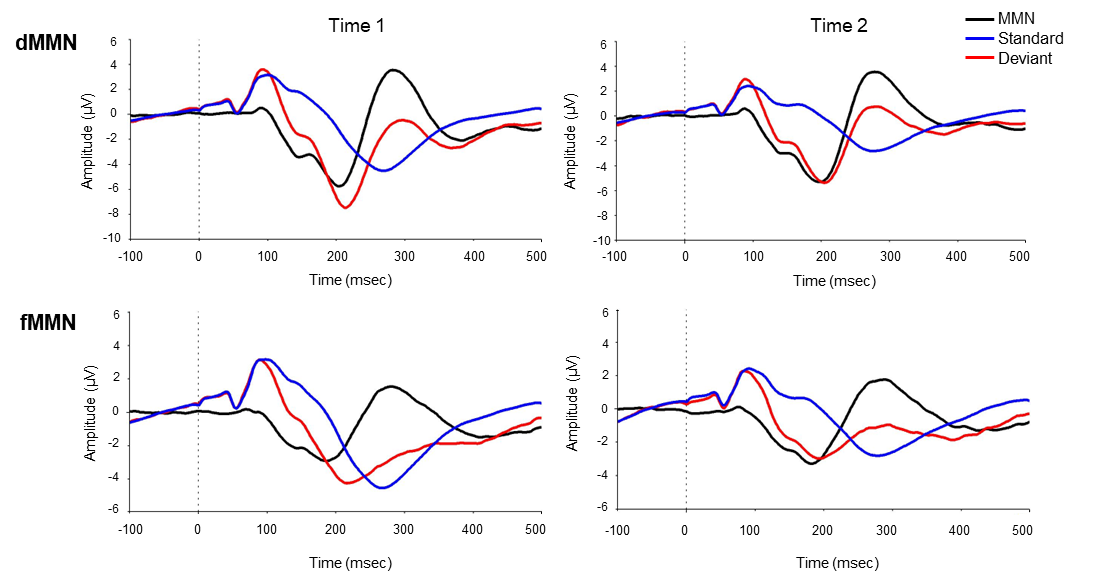
**

(Top) The average waveforms (N = 67) for amplitude (μV) of duration mismatch negativity (dMMN; black), standard condition (blue), and deviant condition (red) at Fz at Time 1 (left) and Time 2 (right).

(Bottom) The average waveforms (N = 67) for amplitude (μV) of frequency mismatch negativity (fMMN; black), standard condition (blue), and deviant condition (red) at Fz at Time 1 (left) and Time 2 (right).

**Supplementary Table 1** Detailed EEG measurement data

| Time | EEG index | Condition | Number of epochs  mean ± SD (range) | Peak latency (msec) of grand average MMN waveform | Sleepiness  mean ± SD |
| --- | --- | --- | --- | --- | --- |
| Time 1  (N = 119) | dMMN | standard | 1716.5 ± 116.3 (1240–1800) | 204 | 2.5 (1.1) |
|  |  | deviant | 190.0 ± 13.4 (139–200) |  |  |
|  | fMMN | standard | 1726.8 ± 92.7 (1351–1798) | 184 | 2.5 (1.0) |
|  |  | deviant | 191.4 ± 10.7 (139–200) |  |  |
|  | ASSR | - | 187 ± 20.6 (113–200) | - | 2.8 (1.1) |
| Time 2  (N = 67) | dMMN | standard | 1715.3 ± 153.4 (1129–1800) | 197 | 2.7 (1.1) |
|  |  | deviant | 189.3 ± 20.2 (103–200) |  |  |
|  | fMMN | standard | 1735.1 ± 106.3 (1277–1799) | 182 | 2.7 (1.1) |
|  |  | deviant | 192.9 ± 11.7 (137–200) |  |  |
|  | ASSR | - | 183.9 ± 23.3 (111–200) | - | 3.0 (1.0) |

Abbreviations: EEG, electroencephalography; dMMN, duration mismatch negativity; fMMN, frequency mismatch negativity; ASSR, auditory steady-state response; SD, standard deviation.

Note: Regarding the longitudinal EEG data, the peak latency (msec) of the grand average waveforms and the mean ± standard deviation (range) of each number of averaged epochs, sleepiness are shown. Subjective sleepiness was measured from 1 to 7 using Stanford Sleepiness Scale.

**Supplementary Table 2** Sex differences of EEG indices

| Time | EEG index | Male (N = 34)  Mean ± SD | Female (N = 33)  Mean ± SD | independent t-tests |
| --- | --- | --- | --- | --- |
| Time 1 | dMMN amplitude (μV) | −5.15 (2.53) | −4.94 (2.85) | *t*_65_ = −0.31, *p* = 0.75 |
|  | fMMN amplitude (μV) | −2.57 (2.08) | −2.80 (1.65) | *t*_65_ = 0.51, *p* = 0.61 |
|  | ASSR ERSP (dB) | 0.99 (0.91) | 1.31 (1.47) | *t*_65_ = −1.08, *p* = 0.29 |
|  | ASSR ITC | 0.27 (0.11) | 0.30 (0.12) | *t*_65_ = −0.90, *p* = 0.37 |
| Time 2 | dMMN amplitude (μV) | −4.85 (2.00) | −4.77 (2.21) | *t*_65_ = −0.17, *p* = 0.87 |
|  | fMMN amplitude (μV) | −2.85 (1.76) | −3.07 (2.05) | *t*_65_ = 0.46, *p* = 0.65 |
|  | ASSR ERSP (dB) | 1.09 (1.41) | 1.20 (1.33) | *t*_65_ = −0.34, *p* = 0.74 |
|  | ASSR ITC | 0.28 (0.13) | 0.28 (0.13) | *t*_65_ = −0.16, *p* = 0.88 |
| Change | dMMN amplitude (μV) | 0.29 (2.14) | 0.17 (1.91) | *t*_65_ = 0.24, *p* = 0.81 |
|  | fMMN amplitude (μV) | −0.28 (2.25) | −0.26 (1.98) | *t*_65_ = −0.03, *p* = 0.97 |
|  | ASSR ERSP (dB) | 0.10 (1.66) | −0.11 (1.87) | *t*_65_ = 0.48, *p* = 0.63 |
|  | ASSR ITC | 0.003 (0.16) | −0.02 (0.15) | *t*_65_ = 0.56, *p* = 0.58 |

^*^ indicates *p* < 0.05

Abbreviations: EEG, electroencephalography; dMMN, duration mismatch negativity; fMMN, frequency mismatch negativity; ASSR, auditory steady-state response; ERSP, event-related spectral perturbation; ITC, inter-trial phase coherence; TD, total difficulties; SD, standard deviation.

**Supplementary Table 3** Alterations in SDQ subscales between Time 1 and Time 2 (N = 67)

| Subscales of SDQ | Time 1  Mean ± SD | Time 2  Mean ± SD | Statistics (paired t-tests) |
| --- | --- | --- | --- |
| Emotional symptoms | 0.7 ± 1.0 | 0.8 ± 1.4 | *t*_66_ = −0.92, *p* = 0.36 |
| Conduct problems | 1.2 ± 1.2 | 1.5 ± 1.1 | *t_66_* = −2.04, p = 0.046^*^ |
| Hyperactivity/inattention | 2.9 ± 2.0 | 2.6 ± 2.1 | *t*_66_ = 1.36, *p* = 0.18 |
| Peer problems | 1.4 ± 1.9 | 1.7 ± 1.8 | *t*_66_ = −1.02, *p* = 0.31 |
| Prosocial behavior | 5.9 ± 2.3 | 5.3 ± 2.4 | *t*_66_ = 2.68, *p* = 0.009^**^ |

^*^ indicates *p* < 0.05; ^**^ indicates *p* < 0.01

Bonferroni corrections were not applied because of the supplementary nature of the analysis.

Abbreviations: SDQ, Strengths and Difficulties Questionnaire; SD, standard deviation.

**Supplementary Table 4** Association between changes in SDQ subscales and dMMN amplitude

| Step | *β* | SE | F | adjusted R^2^ | p |
| --- | --- | --- | --- | --- | --- |
| *Change in SDQ emotional symptoms* | |  | 7.41 | 0.23 | 2.5×10^−4**^ |
| Change in dMMN amplitude | 0.43 | 0.06 |  |  | 2.7×10^−4**^ |
| Sex (Girls, 0; Boys, 1) | −0.30 | 0.23 |  |  | 0.009^**^ |
| Follow-up period | 0.25 | 0.02 |  |  | 0.03^*^ |
| *Change in SDQ conduct problems* | |  | 6.09 | 0.07 | 0.02^*^ |
| Change in dMMN amplitude | 0.29 | 0.07 |  |  | 0.02^*^ |
| *Change in SDQ hyperactivity/inattention* | |  | 4.23 | 0.05 | 0.04^*^ |
| Follow-up period | 0.25 | 0.03 |  |  | 0.04^*^ |
| *Change in SDQ peer problems* | |  | 5.16 | 0.06 | 0.03^*^ |
| Sex (Girls, 0; Boys, 1) | −0.27 | 0.40 |  |  | 0.03^*^ |
| *Change in SDQ prosocial behavior* | |  | 4.52 | 0.10 | 0.02^*^ |
| Change in dMMN amplitude | −0.31 | 0.11 |  |  | 0.013^*^ |
| Follow-up period | −0.25 | 0.03 |  |  | 0.04^*^ |

^*^ indicates *p* < 0.05; ^**^ indicates *p* < 0.01

Bonferroni corrections were not applied because of the supplementary nature of the analysis. The statistics show the results of multiple regression analyses with a stepwise method used by changes in each SDQ subscale as the dependent variable and changes in dMMN amplitude, age in months, and follow-up period as independent variables.

Abbreviations: dMMN, duration mismatch negativity; SDQ, Strengths and Difficulties Questionnaire; SD, standard deviation.
